# Technology-Enabled Solutions for Australian Mental Health Services Reform: Impact Evaluation

**DOI:** 10.2196/18759

**Published:** 2020-11-19

**Authors:** Haley M LaMonica, Alyssa Milton, Katharine Braunstein, Shelley C Rowe, Antonia Ottavio, Tanya Jackson, Michael A Easton, Ashlea Hambleton, Ian B Hickie, Tracey A Davenport

**Affiliations:** 1 Brain and Mind Centre The University of Sydney Camperdown Australia; 2 InnoWell Camperdown Australia

**Keywords:** evaluation methodology, mental health, health care reform, technology, mobile phone, community-based participatory research

## Abstract

**Background:**

Health information technologies (HITs) are becoming increasingly recognized for their potential to provide innovative solutions to improve the delivery of mental health services and drive system reforms for better outcomes.

**Objective:**

This paper describes the baseline results of a study designed to systematically monitor and evaluate the impact of implementing an HIT, namely the InnoWell Platform, into Australian mental health services to facilitate the iterative refinement of the HIT and the service model in which it is embedded to meet the needs of consumers and their supportive others as well as health professionals and service providers.

**Methods:**

Data were collected via web-based surveys, semistructured interviews, and a workshop with staff from the mental health services implementing the InnoWell Platform to systematically monitor and evaluate its impact. Descriptive statistics, Fisher exact tests, and a reliability analysis were used to characterize the findings from the web-based surveys, including variability in the results between the services. Semistructured interviews were coded using a thematic analysis, and workshop data were coded using a basic content analysis.

**Results:**

Baseline data were collected from the staff of 3 primary youth mental health services (n=18), a counseling service for veterans and their families (n=23), and a helpline for consumers affected by eating disorders and negative body image issues (n=6). As reported via web-based surveys, staff members across the services consistently *agreed* or *strongly agreed* that there was benefit associated with using technology as part of their work (38/47, 81%) and that the InnoWell Platform had the potential to improve outcomes for consumers (27/45, 60%); however, there was less certainty as to whether their consumers’ capability to use technology aligned with how the InnoWell Platform would be used as part of their mental health care (11/45, 24% of the participants *strongly disagreed* or *disagreed*; 15/45, 33% were *neutral*; and 19/45, 42% *strongly agreed* or *agreed*). During the semistructured interviews (n=3) and workshop, participants consistently indicated that the InnoWell Platform was appropriate for their respective services; however, they questioned whether the services’ respective consumers had the digital literacy required to use the technology. Additional potential barriers to implementation included health professionals’ digital literacy and service readiness for change.

**Conclusions:**

Despite agreement among participants that HITs have the potential to result in improved outcomes for consumers and services, service readiness for change (eg, existing technology infrastructure and the digital literacy of staff and consumers) was noted to potentially impact the success of implementation, with less than half (20/45, 44%) of the participants indicating that their service was ready to implement new technologies to enhance mental health care. Furthermore, participants reported mixed opinions as to whether it was their responsibility to recommend technology as part of standard care.

## Introduction

### Mental Health Services Reform

Despite more than two decades of effort toward mental health services reform, the Australian system remains to be fraught with shortcomings, including service fragmentation [1], limitations in access [2], and deficient accountability based on outcomes [3]. With an eye toward system reform, the Fifth National Mental Health and Suicide Prevention Plan [4] specifically highlights the need to foster and facilitate enablers for effective system performance and improvement. To that end, health information technologies (HITs) are increasingly being recognized as a way to support and drive mental health services reform, enabling the delivery of evidence-based interventions via the internet to complement or augment traditional face-to-face and existing web-based services to improve health outcomes [5]. For example, cognitive behavioral therapy (CBT) approaches have been incorporated into several apps and websites, including MoodMission [6] and CBT-i Coach [7], to help consumers better self-manage their health and well-being, provide psychoeducation about areas of concern or difficulty, and enhance traditional face-to-face care.

The results of a recent systematic review highlighted the benefits of HITs on the quality and efficacy of health care, partly by facilitating adherence to guidelines or protocol-based care with the aid of embedded electronic decision support functions [8]; however, several facilitators and barriers have been identified that may impact the success of implementation [9]. As summarized thoroughly in LaMonica et al [10], service-level factors include negative staff attitudes; staff members’ resistance to change; and changes to work practices, such as increased workload [11,12], as well as the importance of leadership from senior organizational and local service management to champion the HIT [12,13]. In relation to health professionals, co-designing and configuring the HIT to fit their needs helps to foster buy-in and acceptance [12-14]. Furthermore, successful implementation is facilitated by effective education and training of health professionals, which nurtures self-efficacy and capacity in the context of continuous on-the-ground support [12-15]. The involvement of consumers with lived experience and their families in the co-design process and the consideration of consumer preferences for and disparities in the use of technology are also critical factors for successful implementation. Finally, the adaptability, flexibility, and fit of the technology for the service and its model of care [14,15] should be considered.

### Rapid and Iterative Evaluation and Refinement of HITs

As Mohr et al [16] outline in their Accelerated Creation-to-Sustainment model, when implementing an HIT, it is crucial to evaluate and optimize usability, acceptability, and effectiveness to ensure that it meets the clinical objectives to facilitate successful implementation in the service. Traditional clinical science approaches to the development and implementation of interventions rely on a linear process, including basic science, intervention creation or adaptation, efficacy testing in both research and clinical settings, effectiveness research in community settings, and dissemination [17]. Although the outcomes of each step in this process are indeed valuable, this progressive, staged model can result in delays of up to 17 years for research translation into clinical practice [18]. In contrast, Mohr et al [16] argue against time-consuming pilot and/or clinical trials and rather highlight the superior benefits to be gained from examining the challenges associated with optimization of both the HIT and the implementation plan within a target clinical setting.

As explained in our implementation science strategy [10], our group employs evaluative processes to continuously design, develop, and refine HIT-enabled solutions during implementation. In this case, an HIT-enabled solution refers both to the HIT as well as to the service model in which it is embedded [19]. Our iterative approach ensures that the HIT-enabled solution is adapted for the changing needs of the stakeholders, including consumers with lived experience and their supportive others, as well as the service based on real-world feedback from target users. It is our belief that gathering feedback from all user groups as they utilize the HIT as part of routine clinical practice will facilitate its iterative refinement and optimization and identify the required workforce and structural service-level changes that are required to improve access to and the delivery of quality care. We expected feedback to include technical difficulties as well as comments in relation to user experience and clinical aspects of both the HIT and the associated service model. Importantly, it is generally accepted in digital mental health research that HITs will be iteratively designed, developed, and refined during implementation, with the ultimate aim of integrating an optimized yet adaptable HIT within a service, such that it is seen as a vital piece of standard care, enabling and maintaining ongoing service improvement and system reforms.

### The InnoWell Platform

In 2017, the Australian Government Department of Health (DOH) and InnoWell (a joint venture between the University of Sydney and PwC, Australia) entered into a 3-year funding agreement to deliver Project Synergy (2017-2020). Through a series of collaborative research trials, Project Synergy’s objective is to develop and implement innovative HITs (including the InnoWell Platform) to enable improved mental health service delivery in Australia, facilitating better outcomes for people with lived experience, supportive others, health professionals, and service providers [9,20]. The funding agreement provided for the establishment of a research and development group as well as a product and technology group for the development of the InnoWell Platform.

As described in detail by Davenport et al [21], the co-designed InnoWell Platform was developed through Project Synergy (by InnoWell) to collect, score, store, and report clinical data back to consumers and their health professionals to promote person-centered care, self-management, early intervention, shared decision making, and routine outcome monitoring (Textbox 1, which is a variation of the description of the InnoWell Platform previously published by Hickie et al [9]). All consumers presenting for care to a service utilizing the InnoWell Platform as part of standard service delivery are offered the opportunity to use the InnoWell Platform. The InnoWell Platform utilizes multiple sources of information to develop a comprehensive understanding of a consumer’s needs and to track progress over time, including web-based self-reported psychometric measures assessing a range of biopsychosocial domains (ie, psychological distress, suicidal thoughts and/or behaviors, social and occupational functioning, depressed mood, sleep-wake cycle, social connectedness) from both consumers and their health professionals as well as objective behavioral data collected via third-party integrations (eg, Fitbit). The multidimensional assessment results are reviewed collaboratively by the consumers and their health professionals to promote shared decision making and collaborative care and to facilitate routine outcome monitoring, clinical review, and coordinated care to ensure that all consumers receive the right care, first time.

**Description of the InnoWell Platform as listed on the Australian Register of Therapeutic Goods (ARTG ID 315030; software as a medical device, class 1).**

“The InnoWell Platform is a customisable digital tool that assists assessment, monitoring and management of mental health issues, and maintenance of wellbeing. It does this by collecting personal and health information from consumers and their service providers. This information is stored, scored, and reported back to consumers and their health professionals to promote collaborative care. The clinical content is determined in collaboration with the service provider who invited the consumer to use the Platform. Importantly, the Platform does not provide stand-alone medical or health advice, diagnosis, or treatment. Instead, it guides and supports (but does not direct) consumers and their health professionals to decide what may be suitable care options. Importantly, all care aligns with the existing clinical governance (eg. policies and procedures) of the service provider.”

### Objectives

The primary objective of this study is to gather data through web-based surveys, semistructured interviews, and workshops with staff from the mental health services implementing the InnoWell Platform (eg, health professionals, service managers, and administrators) to evaluate and monitor the impact of embedding the HIT as part of standard service delivery, including (1) digital literacy and competence of the service staff in relation to implementation of the HIT in the service; (2) changes in the service associated with implementation of the HIT-enabled solution; and (3) the quality, usability, and acceptability of the solution. Importantly, the baseline data related to the design, development, and implementation of the InnoWell Platform as part of standard care in Australian mental health services are used to inform the ongoing development of both the HIT as well as the service model in which it is embedded, including implementation processes, for improved user adoption and future sustainment. Additional evaluation data will be captured longitudinally via service audits, user testing, and observational logs; however, reporting on these findings is beyond the scope of this paper.

## Methods

### Study Design

This is a prospective study employing web-based surveys, semistructured interviews, and workshops to identify potential barriers and facilitators for the implementation of an HIT (ie, the InnoWell Platform) and to measure the ongoing impact of implementing the HIT-enabled solution in participating mental health services within Australia. Importantly, before initiating the impact evaluation, the InnoWell Platform is configured for each participating service, ensuring that the solution meets the needs of all end users, from people with lived experience and their supportive others accessing care through to health professionals, service managers, and administrators, using well-established research and development co-design methodologies (such as service mapping, participatory design, knowledge translation, user testing, and rapid prototyping) [9,10,20-22].

### Participating Services and Participants

All staff involved in the implementation of the InnoWell Platform in the service, including health professionals, service managers, and administrators, from *headspace* centers in Port Macquarie, Coffs Harbour, and Lismore; the Butterfly Foundation’s National Helpline; and Open Arms–Veterans and Families Counselling (Sydney) were invited to participate in the impact evaluation study. Staff from the funding and/or governing bodies of the services (ie, service providers and/or primary health networks [PHNs]) who were associated with implementation were also invited to participate. This wide-ranging recruitment is critical to ensure that data are captured from stakeholders at all levels of participating services. Information about research activities, including web-based surveys, semistructured interviews, and workshops, was distributed via email to eligible participants by an implementation officer embedded within the services. In some services, awareness of the impact evaluation was also generated by the display of posters and flyers with details about the research activities. To avoid any perceived coercion, recruitment was passive such that a potential participant needed to contact a member of the study team (using details provided on all study advertisements) who then forwarded the Participant Information Sheet and Consent Form. This study was voluntary, and the participants chose to participate in as many or as few of the research activities as they chose.

### Participant Procedures

Qualitative and quantitative data were collected via the web-based data capture application, REDCap (Vanderbilt University; Multimedia Appendix 1), semistructured interviews (Textbox 2), and a workshop (Textbox 3) with staff at participating services at baseline (ie, preimplementation of the InnoWell Platform). The methods were aligned with the study objectives. Specifically, digital literacy and competence were explored through both web-based surveys and semistructured interviews. As this is reported at the individual level, it was not included in the workshop agenda. All methods were used to investigate the potential quality, usability, and acceptability of the InnoWell Platform as well as the anticipated impact of its implementation on the service. Unique to the semistructured interviews were questions regarding education and training topics that may be of use to the participants and to the consumers of their respective services.

**Excerpt of the baseline impact evaluation semistructured interview questions.**

Topic: Use of the InnoWell Platform

Now, tell me what you think about the implementation of the InnoWell Platform in your service.

- What do you anticipate the impact to be on your practice (eg, more resources for consumers, increased collaboration with consumers regarding treatment planning, increased engagement in care from consumers, changes to efficiency of care, increased case load)?
- Why do you think this change will happen? How do you think this will happen?
- What do you anticipate the impact to be on your service (eg, more resources for consumers, improved team decision making, reduced wait times, improved access)?
- Why do you think this change will happen? How do you think this will happen?
- What do you anticipate the impact to be on your consumers (eg, more resources for consumers, improved ability to self-manage care, increased engagement in care from consumers)?
- Why do you think this change will happen? How do you think this will happen?

**Excerpt of the baseline impact evaluation workshop agenda.**

Topic: Implementation within your service

Group discussion

- When thinking about the implementation of the InnoWell Platform in your service, what do you think will work well for you, the service, and/or your consumers?
- Why do you think this will work well?
- How do you think this will impact you, the service, and/or your consumers?
- What do you see as the value of this change/impact for you, the service, and/or your consumers (eg, time savings, financial savings, etc)?
- What might facilitate (eg, functionality, usability, sufficient training, necessary hardware, etc) the use of the solution for you, the service, and/or your consumers?
- What do you worry will
  not
  work well for you, the service, and/or your consumers?
- Why do you think this will be problematic?

These data collection methods were designed specifically for the purposes of this study and were not piloted before use. Importantly, before engaging in this study, all participants had received training on the InnoWell Platform, including both its functionality and clinical benefits. The participant procedures are repeated every 3 months for the duration of the implementation, the length of which is determined and agreed upon by InnoWell and the participating services; however, reporting on the longitudinal impact evaluation data is outside the scope of this paper.

A template of the web-based survey questions is provided in Multimedia Appendix 1. The surveys were adapted from an eHealth readiness scale developed by Phillips et al [23,24] to assess the readiness of health care teams to effectively and efficiently deliver care using HITs. Semistructured interview questions (Textbox 2) and the workshop agenda (Textbox 3) were designed to identify potential barriers and facilitators to successful implementation not previously uncovered during the preimplementation co-design process (LaMonica et al [10] provide information on the phased implementation strategy). In addition, the longitudinal collection of these data throughout implementation will facilitate revisions over time on the basis of a retrospective review and constructive feedback from services in which the InnoWell Platform is implemented and from the consumers who engage with the solution.

### Data Analysis

The participant sample size is limited by the number of service staff within each mental health service; however, appropriate statistical analyses were conducted and reported based on the sample size for each service, and only aggregate data across services were used for the multidimensional statistical analyses.

Descriptive statistics were used to analyze all aspects of the web-based survey data. Given that the overall sample size was small (n=50), response options were collapsed for some analyses, combining *strongly agree* and *agree* as well as *strongly disagree* and *disagree*. Bivariate analyses using Fisher exact tests were used to evaluate group differences based on participating services, and a reliability analysis was conducted to evaluate the internal consistency of the web-based survey. The alpha level was <.05. SPSS version 24 (IBM Corp) was used for all analyses.

As only one workshop was conducted, a basic content analysis of the scribe notes was conducted by 2 researchers (HL and KB) to identify key service-level barriers and facilitators to implementation. In accordance with the qualitative data analysis processes used by our group [9,20,22], references were tallied, and those references with 3 or more independent tallies were considered to be a consistent theme. Semistructured interviews were audiorecorded, transcribed, and anonymized. Interpretation of the qualitative data from the semistructured interviews followed established thematic techniques [25]. Transcripts were reviewed by 2 research health professionals (HL and AM) to develop a coding framework outlining all key concepts. Transcripts were coded in NVivo 12 software using this framework. The coding followed an established iterative process of reading, coding, exploring the pattern and content of coded data, reflection, and discussion. Similarities and differences in opinion were examined, and differences were resolved through discussions to reach consensus on the coding framework. Themes were then organized by implementation barriers and facilitators for each identified group: (1) consumers accessing the service for support, (2) health professionals working at the service, and (3) the service. The research health professionals checked the themes against each other and back with the original transcripts to ensure that all relevant references had been collated. This process resulted in a thematic framework that was internally coherent and consistent.

### Ethics

This research required multiple ethics approvals by various human research ethics committees (HRECs) because of the diverse organizational structures governing each of the mental health services involved in the impact evaluations. The governing bodies of some mental health services required applications to be submitted through their own internal HRECs (ie, the Department of Defence and Veterans’ Affairs HREC project number 056-18), whereas others preferred that the required applications be submitted through the University of Sydney HREC (project numbers 2018/849 and 2018/962).

## Results

### Participants

A total of 50 staff members from 3 *headspace* centers (Port Macquarie, Coffs Harbour, and Lismore; n=18), Butterfly Foundation’s National Helpline (n=8), and Open Arms–Veterans and Families Counselling (Sydney; n=24) who were trained to use the InnoWell Platform as part of standard service delivery consented to participate in the impact evaluation study. As this study was voluntary, those who consented were not mandated to complete any of the research activities, which resulted in differing rates of participation. Most participants (47/50, 94%) completed the web-based survey. In addition, most participants from Open Arms–Veterans and Families Counselling (17/24, 71%) also participated in the workshop and thus were not inclined to engage in a semistructured interview. In contrast, the variable work schedules of the staff at the Butterfly Foundation’s National Helpline were more conducive to participation in the survey (6/8, 75%) and a semistructured interview (3/8, 38%) as opposed to a workshop. With regard to the *headspace* centers, participants were heavily engaged in the co-design of the InnoWell Platform (studies by LaMonica et al [10] and Davenport et al [21] provide more details) as well as education and training sessions in relation to how to use the InnoWell Platform simultaneously with this study and noted that they did not have the capacity to engage in the semistructured interviews. In addition, given the geographic spread of the participants in these centers, a group-based workshop was not feasible; therefore, use of the web-based surveys was key to ensure participation from this group (18/18, 100%). Importantly, despite variable participation rates in each research activity, methodological triangulation ensured that data capture was comprehensive and inclusive of all relevant stakeholders.

### Web-Based Survey Outcomes

A total of 47 participants completed the web-based survey at baseline, representing diverse roles in the services (Table 1).

**Table 1:** Participants’ (n=47) roles across services.

Role	Participants, n (%)
General psychologist	12 (26)
Social worker	10 (21)
Counselor	6 (13)
Service managers and administrators	4 (9)
Mental health nurse	2 (4)
Youth worker	2 (4)
Dentist	1 (2)
General practitioner	1 (2)
Other	9 (19)

Cronbach alpha was calculated for the 27 Likert-scale survey questions and indicated that the survey was acceptably reliable (α=.86). As shown in Table 2, the participants endorsed considerable value associated with using technology as part of their work and consistently *agreed* or *strongly agreed* that HITs have the potential to improve outcomes for consumers. Importantly, most respondents indicated that the proposed technology (ie, the InnoWell Platform) was appropriate for their services’ consumers and that service staff were willing to implement the HIT for its intended purpose. Importantly, there was no notable difference in participants’ perceptions of the role of the technology in mental health care or the appropriateness of the HIT on the basis of the participating service (Table 2).

**Table 2:** Differences in aggregated web-based survey responses from participants based on the participating service.

Question^a^	Strongly disagree or disagree, n (%)	Neutral, n (%)	Strongly agree or agree, n (%)	*P* value
I see the benefit of using technology as part of my work (n=47)	7 (15)	2 (4)	38 (81)	.55
How do you feel about this statement: “My organisation is making the best use of technology for mental health care” (n=47)	12 (26)	8 (17)	27 (57)	.48
Technology has made mental health care change too fast (n=46)	21 (46)	19 (41)	6 (13)	.58
My service feels it is part of our professional role to actively recommend technologies for mental health care and provide assistance to consumers (n=46)	6 (13)	15 (33)	25 (54)	.50
Our consumers’ capability to use technology is aligned with how technology will be used in their mental health care (n=45)	11 (24)	15 (33)	19 (42)	.39
My service has a work culture that actively encourages the integration of technologies (n=45)	4 (9)	17 (38)	24 (53)	.83
My service’s policies reflect a belief that technologies can improve consumer outcomes by providing more efficient and effective services (n=45)	4 (9)	14 (31)	27 (60)	.88
On average, consumers appear to have a positive experience with using technology as part of their mental health care (n=45)	5 (11)	19 (42)	21 (47)	.21
My service is ready to implement new technologies to enhance mental health care (n=45)	14 (31)	11 (24)	20 (44)	.98
The proposed technology (ie, InnoWell Platform) is appropriate for the consumers who are cared for in the service (n=45)	8 (18)	12 (27)	25 (56)	.08
There is a willingness within the service to implement the technology for its intended purpose (n=45)	2 (4)	12 (27)	31 (69)	.47

^a^Percentage totals for each row do not always sum up to 100% due to rounding error.

Most participants indicated they were *very* *aware* (7/27, 26%) or *somewhat aware* (18/27, 67%) of HITs that support or directly provide mental health care, indicating a wide range of sources for learning about these types of technologies (Table 3).

**Table 3:** Participants’ (n=47) sources of learning about technologies for mental health care.

Source	Participants, n (%)
Colleagues	34 (72)
Personal research	26 (55)
Professional development organizations	26 (55)
Training sessions conducted by the service	25 (53)
Websites	22 (47)
Consumers	20 (43)
Manager	16 (34)
Social media	15 (32)
Friends and family	13 (28)
Supervisor	13 (28)

The frequency with which participants tried different technologies as part of their role was highly variable (Never: 6/47, 13%; Not very often: 16/47, 34%; and Sometimes: 25/47, 53%). The primary reasons for not trying technologies as reported by the 22 participants who responded “Never” or “Not very often” included a lack of time to experiment (6/22, 27%), technological limitations of the service (5/22, 23%), and a lack of interest from consumers in relation to using technology as part of their mental health care (2/22, 9%), with the participants being given the option to choose all reasons that applied for this question.

### Semistructured Interviews

**Figure 1:**
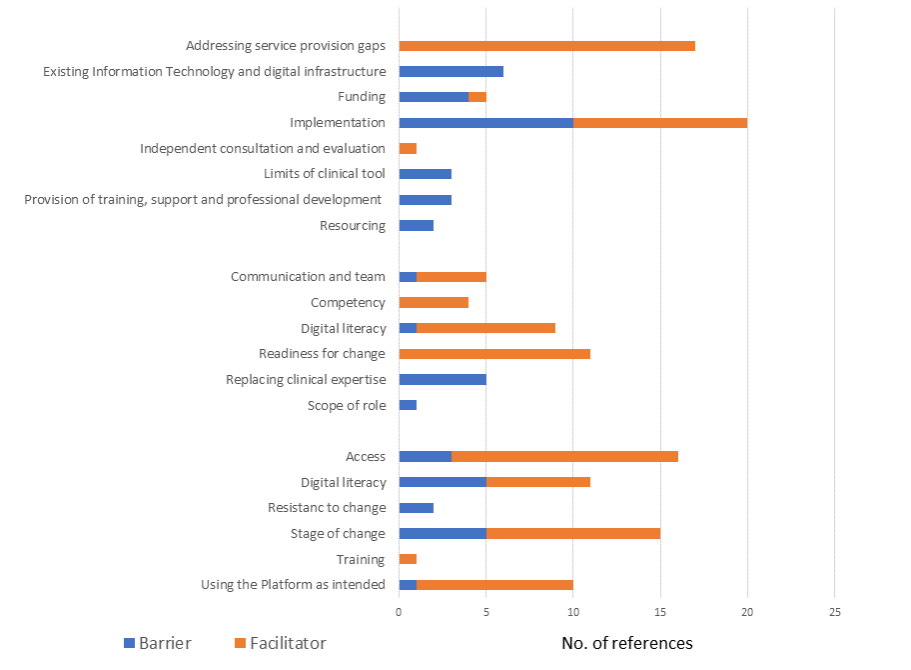
Codes and themes.

Given the 16-hour workforce schedule (7 days a week), the 60-min semistructured interviews (n=3) were the preferred method of providing qualitative feedback for health professionals from the Butterfly Foundation’s National Helpline. Qualitative themes were organized to reflect the barriers and facilitators of the implementation of the InnoWell Platform within the service. These barriers and facilitators contained 3 groups: (1) *consumer*, which included implementation factors (barriers or facilitators) that impacted the consumers of the service; (2) *health professional*, which included implementation factors that impacted the health professionals working at the service; and (3) *service*, which included implementation factors that were considered at a service level. The barrier and facilitator themes for each group (*consumer, health professional*, and *service*) are presented in Figure 1, with illustrative quotes provided in Multimedia Appendix 2. As displayed in Figure 1, facilitators to implementation were referenced more frequently (52 references) than barriers (39 references). A reference refers to the selection of content from the interviews that has been coded. References can be coded under more than one theme; in this case, they are counted as more than one reference. It should be noted that the sum of the references of the parent theme (ie, barriers) reflects the number of unique references for that theme. As references may have been coded to more than one subtheme (ie, digital literacy and readiness for change), the sum of references for each subtheme will not equate to the sum of references for the parent theme.

#### Consumer Barriers and Facilitators

For consumers accessing the service, the most commonly reported barrier and facilitator themes are related to *access* and *staging.* In terms of *access*, all participants felt that technology could promote better access for consumers to services, particularly in rural and regional areas, but such technologies might not be accessible in certain services, such as in hospital settings. The theme of *digital literacy* also overlapped with the theme relating to *access,* with all participants feeling that the InnoWell Platform might be more accessible to (and adopted by) young people, who were digital natives, whereas those “...who are a bit older...don’t use internet” and “...they would need some time to adapt” (interview 1). One participant felt the provision of “some sort of *training* that is accessible, probably free, that’s easy and not time consuming” would be important for consumers to help them to use the InnoWell Platform (interview 1). *Staging* related to both the consumer’s clinical stage of help-seeking and their stage of change (as per the transtheoretical model [26]), with potential benefits noted for both consumers who had previously received treatment and those who had not yet sought help as it might be “...a lot less invasive or intrusive or confronting” (interview 3).

*Using the InnoWell Platform as intended* was a common facilitator theme but was only referenced once as a barrier. All participants envisioned that technology could complement the support a consumer received by being a “...one-stop shop for resources” (interview 3) and by being used as a communication tool for services and for seeking help. However, 1 participant cautioned that it was important to be aware that some may “...twist things to make it appear they’re okay to themselves” (interview 1).

#### Health Professional Barriers and Facilitators

Although the main implementation barrier identified for health professionals generally related to concerns that the tool could *replace clinical expertise,* participants denied this as a personal concern, noting that the InnoWell Platform may “...enrich the counseling side of things” (interview 1), “...free up the counselors” (interview 2), or “...supplement a counseling practice, in so far as helping them support people ahead of the contact with the therapist” (interview 3). Interestingly, all participants commented that they felt that they had a high level of *digital literacy* and *competency* in their role and were *ready for change*; however, 1 participant noted that the use of new technologies was outside the *scope of their role* in the service. Good *communication and team* environments were seen as vital to implementation. This feedback was not only from service management but also from colleagues as it created a culture of support, with one participant highlighting “I think it’s everybody. It’s just the culture” (interview 3).

#### Service Barriers and Facilitators

At the service level, the main barriers were associated with the interrelated themes of *implementation*, *existing information technology (IT) infrastructure*, *funding*, and *resourcing*. The first 2 participants had major concerns about the current funding situation, the “clunky” (interview 1) existing infrastructure—highlighting that “...we are pretty over extended as it is” (interview 1). All participants felt that technology could *address service gaps* “...quite well, especially (for) those who live regionally and (are on) waitlists” (interview 1). In fact, *addressing service gaps* was the main facilitator theme at the service level highlighted by participants. The third interview, however, took place immediately after the introduction of new IT systems, commencement of new funding, and the early implementation of the InnoWell Platform. This participant had very positive views of *implementation* and felt that the service was being quite “innovative...trying to include or integrate technology as part of what they offer...” (interview 3). Positive views were attributed to the InnoWell Platform being supported through proper resourcing, implementation, planning, and training.

### Workshop Themes

Owing to highly variable work schedules (ie, 16-hour schedule) and the distance between participating services, the baseline workshop was only conducted at Open Arms (Sydney) with 17 participants, including health professionals, service managers, and administrators. The 90-min workshop was titled, *Anticipating what is to come: Implementation of the InnoWell Platform within the Open Arms (Sydney) Centre*, and the agenda (Textbox 2) focused on identifying potential barriers and facilitators to successful implementation of the technology.

The primary theme (15 tallied references) that emerged from the workshop related to an immediate need to clarify or establish procedures to facilitate implementation of the HIT and to allocate responsibility to staff for new tasks resulting from that implementation. For example, participants were asked to clarify the following:

- “What are the (consumer) allocation processes within the InnoWell Platform?”
- “How is the decision made whether the InnoWell Platform is offered to individuals or not?”
- “How will (the InnoWell Platform) interface with national intake?”
- “How are we keeping track of how many (consumers) are being offered and saying yes/no?”
- “What happens when consumers leave the service but re-engage again a few months later?”
- “Who will assess eligibility for the service?”

At the end of the workshop, the service leadership agreed to put in place the following mitigation strategies before implementation: the development of a tracking system to log consumer uptake of the InnoWell Platform, clarification of eligibility screening criteria and procedures, updated intake assessment requirements for consumers who had previously accessed care through the service, data requirements for the electronic medical records, timelines for allocating consumers who completed their intake in the InnoWell Platform to a health professional, standard procedures for responding to moderate-to-high levels of suicidal thoughts and/or behaviors identified in the InnoWell Platform, and procedures for contacting consumers with incomplete data in the InnoWell Platform. No other themes emerged from the content analysis.

## Discussion

### Benefits of the Evaluation

HIT-based innovation efforts are increasingly recognized as a way to support and drive mental health services reform for improved quality and clinical outcomes. Integral to this process is the need to continuously evaluate the quality, acceptability, and usability of these HIT-enabled solutions, encompassing both the technology itself and the service models in which they are implemented. Evaluation provides the necessary information to further develop and refine the HIT as well as to understand the interactions with and impact of the HIT by the mental health service staff (health professionals, service managers, and administrators) to reform or refine the service model. Our impact evaluation, including web-based surveys, semistructured interviews, and a workshop, facilitated the rapid iterative development of the HIT and the service model in which it was embedded and is consistent with a usability engineering approach to software evaluation.

Importantly, our findings highlight the value of our impact evaluation methods as a means to discover potential barriers to implementation. Although our group employs a 4-phase, evidence-driven implementation science strategy designed to systematically guide the successful implementation of HITs in mental health services [10], several potential barriers remained unrecognized and unmitigated. As part of this strategy, all participating sites are included in a thorough co-design process, often cited as a primary strategy to facilitate successful implementation [11-13], to ensure the appropriateness and acceptability of the HIT-enabled solution for the service, including consumers, health professionals, and service administrators. Despite using established strategies to mitigate potential barriers to implementation, our data highlight several factors yet to be addressed. For example, specifically as a result of this research, service-level processes were developed by a participating service before the implementation of the HIT-enabled solution with the aim of minimizing workflow disruptions for impacted staff, a well-known risk to successful implementations. This included establishing procedures and allocating responsibility to staff for new tasks resulting from the implementation, such as determining the eligibility for access to the InnoWell Platform based on desired clinical services (eg, individual therapy vs family counseling), monitoring consumers’ engagement with the InnoWell Platform (ie, completion of the web-based multidimensional assessment) to determine when they are ready to be allocated to a health professional, and overseeing the allocation of consumers to a health professional based on the clinical need identified through the InnoWell Platform.

The unique use of methodological triangulation (ie, mixed methods) ensured that data collection was comprehensive and inclusive of all stakeholders to drive an enhanced understanding of the potential impacts of implementation. The triangulation of data has previously been used to evaluate the implementation of HITs among health service professionals [27,28]. This method not only allows for a richness of qualitative and quantitative data capture [29] but also ensures that outcomes are measured at all levels of the health services, including all key stakeholders. In relation to the latter, the use of mixed methods in this study was invaluable as it facilitated the participation of staff members from different service models (ie, a web-based helpline operating on a 16-hour schedule vs traditional face-to-face counseling services operating during normal business hours), thus ensuring both breadth (via the surveys) and depth (via the semistructured interviews and workshop) of evaluation from a broad range of participants. Not surprisingly, the rate of participation (47/50, 94% of consented participants) was highest for the web-based survey, with participation in the workshops and semistructured interviews being largely dictated by the service model and schedule. The application of methodological triangulation in this case was important to determine whether the InnoWell Platform was likely to be effective and whether further co-design was required to improve the alignment of the HIT with the service’s goals for reform. In future research, the methods will be further strengthened by the inclusion of service audits, user testing, and observational logs.

### Barriers to and Facilitators of Implementation

There was consistent agreement across services regarding the potential benefits of technology as part of mental health care service provision, with most respondents on the web-based surveys indicating that they believed that technologies could improve outcomes by providing more efficient and effective services (27/45, 60% of the participants chose “agree” or “strongly agree”). This aligns with existing literature highlighting the potential for HITs to improve consumer outcomes. For example, several trials have shown that a personal health record can significantly improve outcomes and increase the use of routine preventive medical services [30-32]. A personal health record shifts the management of health data from health professionals and/or services to the consumer, enabling active participation in care. They frequently include decision support tools to help consumers manage chronic health conditions and are able to integrate with other data sources, such as electronic medical records, to support coordinated, person-centered care [33].

It was also agreed that HITs have the potential to improve access, particularly for consumers in regional, rural, and remote areas, and address gaps in service provision. Increasing funding for these types of services also signals a growing acceptance of HIT-enabled models of care [34,35]. Within the health sector, it is agreed that technology can help consumers overcome access barriers, including time constraints, transportation problems, and cost [36]. Consideration of the *digital divide*, however, is critical to ensure that those who may not have easy access to technology (eg, internet, smartphone) or the skills required to use it (eg, older adults) are not excluded from receiving mental health care delivered via HITs. To that end, our results indicate that the digital literacy of consumers is both a potential barrier to and facilitator of implementation, with some respondents questioning whether consumers had the required digital literacy to engage with the InnoWell Platform as part of their care (11/45, 24% of the participants chose “disagree” or “strongly disagree”; 15/45, 33% chose “neutral”; and 19/45, 42% chose “agree” or “strongly agree”). As the study participants worked in services providing care to varied consumer groups in different regions of Australia (urban and regional), it is possible that sociodemographic factors, such as age, gender, and socioeconomic status, may explain the latter finding. Data from a substudy of the Bettering the Evaluation and Care of Health program indicate that there is an inverse relationship between age and internet usage for web-based health information. Furthermore, socioeconomically disadvantaged patients were found to be less likely to use the internet, access health information on the web, or obtain health information related to a condition being managed by their general practitioner [37]. Recommendations to bridge the digital divide include (1) technology subsidies for low-income consumers, (2) user-friendly technologies appropriate for consumers with physical and intellectual disabilities, and (3) demonstrations and training opportunities for consumers who might otherwise not have the opportunity to learn how to use available technologies [38].

The digital literacy of health professionals was also noted to be an important factor to consider during implementation. Although the health sector is rapidly embracing technology as an integral part of effective work practices and service provision, the digital literacy of health professionals may not be sufficient to maximize the potential of HITs. For example, a study of 10,000 nurses in Australia found low levels of use of computer-based applications as well as poor confidence in using such tools. Strikingly, less than 40% of respondents in the study indicated that they “frequently” or “always” used HITs (eg, accessing patient records and results) as part of their work [39]. Furthermore, a recent systematic review found low levels of digital literacy among pharmacists in Australia, Canada, and the United States [40]. To realize the full potential of HITs, investment is urgently needed in the training and professional development of health care staff to ensure competence and confidence in using HITs, the latter of which has shown to improve engagement with technology [41].

Importantly, most participants believed that the InnoWell Platform was appropriate for their consumers (25/45, 56% of the participants chose “agree” or “strongly agree”); however, nearly one-third of the respondents did not believe that their service was ready to implement the HIT (14/45, 31% of the participants chose “disagree” or “strongly disagree”). Participants emphasized the need to establish clear processes for implementation, particularly in relation to how the InnoWell Platform will integrate with or change current workflows, including screening consumers for service eligibility, creating digital case files in existing electronic medical records, allocating consumers to health professionals, and responding urgently to risk identified via the HIT. These concerns align with existing literature, which highlights several factors that can impact organizational readiness to adopt new technologies, including (1) leadership both at the executive and local level to help ensure alignment between the technology and the service mission as well as to foster organizational support for the HIT-enabled solution [12-15], (2) misalignment between conventional service models and workflows with the HIT-enabled solution [42], (3) limitations in the availability of appropriate resources (eg, information and communications technology) and personnel [12,15], and (4) required interoperability with other existing technology systems used within the service [12].

Our results also showed that the readiness of health professionals to adopt this type of HIT as part of their practice may be a key facilitator for implementation. Most respondents indicated a willingness to implement the HIT-enabled solution (31/45, 69% of the participants chose “agree” or “strongly agree”), likely reflecting the benefits of the proceeding co-design process as a means to foster buy-in and acceptance among the health professionals involved in this study. With that being said, staff members’ resistance to change and negative staff attitudes are consistently reported in the literature as potential barriers to implementation [11,12]. Furthermore, HITs can be perceived as impersonal as they reduce the need for face-to-face interactions with consumers [43]. Effective education and training in the context of continuous on-the-ground support is a critical mitigation strategy [12-15]. Similarly, establishing a clear communication strategy to support consistent messaging to staff, service users, and other key stakeholders is key to a successful implementation [12].

### Limitations

This study has some limitations in relation to sample size, which are important to note. In particular, as a product of differing service models, only 3 semistructured interviews were conducted with participants from the Butterfly Foundation’s National Helpline, and only a single workshop was run with participants from Open Arms (Sydney). Although the aim had been to utilize all methodologies across participating services, the consistency in the themes identified suggests that this did not notably skew or impact the findings. Rather, we present formative results that will be further explored in future research projects, both at the outset of new implementations as well as longitudinally, as described in the Future Directions section.

### Future Directions

Future services participating in this impact evaluation research may include services for children and their families, specialist youth mental health services, adult staged-care services, older persons mental health, and general practice, allowing for the collection of evaluation data of greater depth and breadth. Furthermore, as mentioned in the Methods section, the impact evaluation data described herein will be collected quarterly for the duration of the implementations and will be complemented by service audits, user testing, and observational logs. These longitudinal data will (1) facilitate ongoing iterative co-design and refinement of our HIT-enabled service model; (2) provide valuable insights into the impact of the implementation on consumer outcomes, health professional practices, and key service-level performance indicators, such as safety, satisfaction and acceptability, appropriateness, efficiency, accessibility and equity, effectiveness, continuity and coordination, and competence and capability; and (3) support the evaluation of social return on investment (ie, the social, environmental, and/or economic value) of our HIT-enabled service model [44].

### Conclusions

The implementation of HIT-enabled solutions in services is inherently disruptive as they bring change to conventional practice for all stakeholders (eg, health professionals, service managers, and administrators). Despite the extensive co-design methods used in the preimplementation phase [10], our impact evaluation methods allowed for the identification of barriers and facilitators that had not otherwise been uncovered, providing a critical opportunity for mitigation to reduce the potential for implementation failure. Notably, the results of an American Medical Informatics Association Workshop highlighted that failures in the implementation of HITs were largely driven by managerial rather than technical factors [45]. Ongoing collaboration and research and development between researchers and participants (eg, health professionals, service managers, and administrators) to facilitate the iterative co-design and development of the HIT and, perhaps more importantly, the service model in which it is embedded are critically important components for the success of implementation and sustainability for mental health services reform.

## Appendix

Multimedia Appendix 1 Web-based survey template.

Multimedia Appendix 2 Codes and themes.

## Abbreviations

BMC: Brain and Mind Centre
CBT: cognitive behavioral therapy
DOH: Department of Health
HIT: health information technology
HREC: human research ethics committee
IT: information technology
PHN: primary health network
