# Supplementary material for: Technology-Enabled Solutions for Australian Mental Health Services Reform: Impact Evaluation
Source: JMIR Form Res. 2020 Nov 19;4(11):e18759. doi: 10.2196/18759 (PMC7714649; doi:10.2196/18759)
Supplement: Multimedia Appendix 1 [file formative_v4i11e18759_app1.docx]

Multimedia appendix 1. Project Synergy: Impact Evaluation Web-based Survey (Baseline)

1. What is your current age?

- 15 to 19 years
- 20 to 24 years
- 25 to 29 years
- 30 to 34 years
- 35 to 39 years
- 40 to 44 years
- 45 to 49 years
- 50 to 54 years
- 55 to 59 years
- 60 to 64 years
- 65 to 69 years
- 70 to 74 years
- 75 to 79 years
- 80 to 84 years
- 85 years and over

1. What is your gender?
   - Male
   - Female
   - Do not identify has male or female
   - Prefer not to answer
2. What is your primary spoken language?
   - English 🡪 Skip to Q6
   - Other 🡪 Continue to Q4
3. Please specify
   - Mandarin 🡪 Skip to Q6
   - Arabic 🡪 Skip to Q6
   - Cantonese 🡪 Skip to Q6
   - Vietnamese 🡪 Skip to Q6
   - Italian 🡪 Skip to Q6
   - Greek 🡪 Skip to Q6
   - Tagalog 🡪 Skip to Q6
   - Hindi 🡪 Skip to Q6
   - Spanish 🡪 Skip to Q6
   - Punjab 🡪 Skip to Q6
   - Other 🡪 Continue to Q5
4. Please specify
   - Please input
5. Does the main language in which you deliver services differ from your primary spoken language?
   - No 🡪 Skip to Q10
   - Yes 🡪 Continue to Q7
6. What is the main language in which you provide care?
   - English 🡪 Skip to Q10
   - Other 🡪 Continue to Q8
7. Please specify
   - Mandarin 🡪 Skip to Q10
   - Arabic 🡪 Skip to Q10
   - Cantonese 🡪 Skip to Q10
   - Vietnamese 🡪 Skip to Q10
   - Italian 🡪 Skip to Q10
   - Greek 🡪 Skip to Q10
   - Tagalog 🡪 Skip to Q10
   - Hindi 🡪 Skip to Q10
   - Spanish 🡪 Skip to Q10
   - Punjab 🡪 Skip to Q10
   - Other 🡪 Continue to Q9
8. Please specify
   - Please input
9. Are you of Aboriginal or Torres Strait Islander origin?
   - No
   - Aboriginal
   - Torres Strait Islander
   - Both Aboriginal and Torres Strait Islander
   - Prefer not to answer
10. What is your profession (e.g. psychologist, psychiatrist)?
    - Alcohol and Substance Use Clinician
    - Clinical Psychologist (with endorsement)
    - Counsellor
    - Dentist
    - Dietitian
    - General Practitioner
    - General Psychologist
    - Mental Health Nurse
    - Neuropsychologist
    - Nurse
    - Occupational Therapist
    - Occupational Therapy Trainee
    - Provisional Psychologist
    - Psychiatrist
    - Psychiatry Registrar
    - Social Worker
    - Social Worker Trainee
    - Other - please specify? **Added**
11. Is your profession the same as your role (e.g. your profession may be Clinical Psychology, but your role in service may be as a Youth Access Clinician)?
    - Yes 🡪 Skip to Q14
    - No 🡪 Continue to Q13
12. What is your current role at [service]?
    - Aboriginal and Torres Strait Islander Mental Health Worker
    - Alcohol and Substance Use Clinician
    - Case Manager
    - Clinical Psychologist
    - Counsellor
    - Crisis Counsellor
    - Family Counsellor/ Therapist
    - Intake Clinician
    - Peer Support Worker
    - Service Manager
    - Service Administrator
    - Youth Access Clinician
    - Other (please specify)
13. How many years in total have you been practicing in this discipline?
    - Please input
14. How many years have you been practicing in your current role?
    - Please input
15. What is your professional background? (e.g. education and training)

Please select all that apply:

- TAFE Certificate/ Diploma
- University Diploma
- University Degree (e.g. BA, BSc)
- Master’s Degree
- PhD
- Doctorate

1. What is your full time equivalent at this service? In other words, what is the ratio of your total number of paid hours during a work week by the number of working hours in a week (e.g. if you work two, seven-hour days per 35 hour week, your full time equivalent would be 0.4).
   - 0.1
   - 0.2
   - 0.3
   - 0.4
   - 0.5
   - 0.6
   - 0.7
   - 0.8
   - 0.9
   - 1.0
2. I see the benefit of using technology as part of my work.
   - Strongly disagree
   - Disagree to some extent
   - Neither agree nor disagree
   - Agree to some extent
   - Strongly agree
3. Which statement would you say best describes your view about the impact of technology on your service?
   - I am worried that technology’s emphasis on speed and immediacy poses a danger to the accuracy of the care provided to clients
   - I am excited that technology offers the potential for greater-than-ever mental health care
   - I’m conflicted because I feel a mixture of both
   - Not sure
4. How do you feel about this statement: “*My organisation is making the best use of technology for mental health care*”

- Strongly disagree
- Disagree to some extent
- Neither agree nor disagree
- Agree to some extent
- Strongly agree

1. How aware are you of technologies for mental health care?

- Very unaware
- Somewhat unaware
- Neither aware nor unaware
- Somewhat aware
- Very aware

1. How do you learn about technologies for mental health care?

Please select all that apply:

- From my manager
- From my supervisor
- From [service] training sessions
- From professional development organisations
- From my own research
- From different websites
- From social media
- From my colleagues
- From clients
- From friends and family
- Other (please specify)

1. Do you try out different technologies in your service?

- Never
- Not very often
- Sometimes
- Yes, somewhat
- Yes, a great deal
  - If ‘Not very often’ or ‘Never’ 🡪 Continue to Q24
  - If ‘Yes, a great deal’ or ‘Yes, somewhat’ or ‘Sometimes’ 🡪 Skip to Q25

1. Why don’t you try out different technologies in your service more often?

Please select all that apply:

- They still seem too hard
- Don’t have time to experiment
- Technological limitations in my practice/ service
- I just haven’t gotten around to it
- Too expensive
- Tools don’t seem advanced
- I don’t know if I’ll have a use for them
- My clients are not interested in using technologies as part of their mental health care
- Other (please specify)

1. Rate the pace of digital transformation at your service:

- Way too slow
- A little too slow
- About right
- A little too fast
- Way too fast

1. Which of the following words would you use to describe your services approach toward technological innovation?

Please select all the words that apply:

- Ambitious
- Average
- Cautious
- Cutting edge
- Guarded
- Inattentive
- Inconsistent
- Lagging
- Leading
- Negligent
- Noncommittal
- Ordinary
- Patient
- Proactive
- Reactive
- Reluctant
- Slow
- Strategic
- Tracking
- Trailblazing

1. Technology has made mental health care change too fast.

- Strongly disagree
- Disagree to some extent
- Neither agree nor disagree
- Agree to some extent
- Strongly agree

1. How would you rate technology’s impact on mental health care?

- Very negative
- Negative
- Neutral
- Positive
- Very positive

1. How much do you personally agree or disagree with each of the following statements:

|  | Strongly disagree | Disagree | Neutral | Agree | Strongly agree |
| --- | --- | --- | --- | --- | --- |
| I believe I am able to make good use of Internet websites and web applications. | ☐ | ☐ | ☐ | ☐ | ☐ |
| My service has received training in the clinical use of technology. | ☐ | ☐ | ☐ | ☐ | ☐ |
| My service team feels that it is part of our professional role to actively recommend technologies for mental health care and provide assistance to clients. | ☐ | ☐ | ☐ | ☐ | ☐ |
| There is sufficient technological support for using technologies in my service. | ☐ | ☐ | ☐ | ☐ | ☐ |
| My service uses technologies as part of treatment for clients (includes both administrative and clinical technologies). | ☐ | ☐ | ☐ | ☐ | ☐ |
| Our clients’ capability to use technology is aligned with how technology will be used in their mental health care. | ☐ | ☐ | ☐ | ☐ | ☐ |
| My service has a work culture that actively encourages the integration of technologies. | ☐ | ☐ | ☐ | ☐ | ☐ |
| My service’s policies reflect a belief that technologies can improve client outcomes by providing more efficient and effective services. | ☐ | ☐ | ☐ | ☐ | ☐ |
| On average, clients’ appear to have a positive experience with using technologies as part of their mental health care. | ☐ | ☐ | ☐ | ☐ | ☐ |
| My services policies reflect a belief that technologies can improve patient outcomes by providing more efficient and effective services. | ☐ | ☐ | ☐ | ☐ | ☐ |
| The clients treated in my service, on average, have enough digital literacy to use computer technology to locate and understand relevant information. | ☐ | ☐ | ☐ | ☐ | ☐ |
| Clients treated in my service see the value of technologies as part of their mental health care. | ☐ | ☐ | ☐ | ☐ | ☐ |
| The technologies used in my service are up to date. | ☐ | ☐ | ☐ | ☐ | ☐ |
| My service has a positive attitude towards technology. | ☐ | ☐ | ☐ | ☐ | ☐ |
| My service has received training in the administrative use of technologies. | ☐ | ☐ | ☐ | ☐ | ☐ |
| Members of my service are receptive to changes in clinical processes. | ☐ | ☐ | ☐ | ☐ | ☐ |
| My service is ready to implement new technologies to enhance mental health care. | ☐ | ☐ | ☐ | ☐ | ☐ |
| The proposed technology is appropriate for the clients who are cared for in the service. | ☐ | ☐ | ☐ | ☐ | ☐ |
| There is a willingness within the service to implement the technology for its intended purpose. | ☐ | ☐ | ☐ | ☐ | ☐ |

1. Do you provide clinical care to individuals accessing your service?

- Yes 🡪 continue to question 31
- No 🡪 end of questionnaire

1. In the last 2 weeks, to what extent did you employ the following in your usual clinical care:

|  | **N/A** (not applicable to my service)  (0) | **Not at all** (with no clients)  (1) | **Rarely**  (with a small proportion of clients) (2) | **Sometimes** (with about half of clients)  (3) | **Very Often** (with most clients)  (4) | **Always** (all of the time, for all clients) (5) |
| --- | --- | --- | --- | --- | --- | --- |
| Broad, multi-dimensional assessment of needs, beyond mental health signs and symptoms | ☐ | ☐ | ☐ | ☐ | ☐ | ☐ |
| Assessment of clinical stage | ☐ | ☐ | ☐ | ☐ | ☐ | ☐ |
| Match the ‘intensity’ of an intervention to the needs of the individual | ☐ | ☐ | ☐ | ☐ | ☐ | ☐ |
| Change the ‘intensity’ of an intervention to the needs of the individual over the course of care | ☐ | ☐ | ☐ | ☐ | ☐ | ☐ |
| Shared or collaborative decision making with individuals under your care | ☐ | ☐ | ☐ | ☐ | ☐ | ☐ |
| Use tools to monitor and track changes to symptoms or function in individuals under your care for the purpose of treatment planning | ☐ | ☐ | ☐ | ☐ | ☐ | ☐ |
| Assess and proactively respond to suicidal thoughts and behaviours | ☐ | ☐ | ☐ | ☐ | ☐ | ☐ |
