# Supplementary material for: Technology-Enabled Solutions for Australian Mental Health Services Reform: Impact Evaluation
Source: JMIR Form Res. 2020 Nov 19;4(11):e18759. doi: 10.2196/18759 (PMC7714649; doi:10.2196/18759)
Supplement: Multimedia Appendix 2 [file formative_v4i11e18759_app2.docx]

Multimedia Appendix 2. Codes and Themes

| **Barriers (39 references)** | **Facilitators (52 references)** |
| --- | --- |
| ***Individual (14 references)*** | ***Individual (30 references)*** |
| **Access**  “Yeah, and you know certain treatment landscapes may limit the ability to use online services. Say if someone is inpatient may not be availability of using an iPhone. So maybe barriers to access as much as one would want to.” (*Interview 2*) | **Access**  “I guess psychoeducation as well, the brief intervention, online delivery of individual services, like Skype is obviously one thing that a lot of people are doing. Um, I think that’s a really important thing and a really good way of doing things, especially for regional and rural areas, and even just in the city. I know a lot of people preferring Skype because what’s the point of battling traffic, and parking and this and this and that, when you can just jump on and. (*Interview 3*)  “Finances are a big barrier in treatment. So something that’s accessible would actually be a good option.” (*Interview 2*) |
| **Digital Literacy**  “…carers who are a bit older, some of them don’t use internet — they would need some time to adapt.” (*Interview 1*) | **Digital Literacy**  “Younger people are much more tech-savvy and they’re used to being online and connected all the time, just trying out new things. So yeah, definitely age… Maybe their education as well. If they’re well educated, then they’re more likely to be exposed to those kinds of things.” (*Interview 2*) |
| **Staging**  “Yeah and I’m thinking even longevity of illness, maybe this would be more likely to be used by someone who has received treatment before or is kind of savvy and knows what’s available. Whereas for somebody who is in the initial stage of wanting help and treatment maybe not so much.” (*Interview 1*) | **Staging**  “Participant: So people who might before have had no intervention or no access to any kind of help or support, then actually having it because they’re not necessarily ready to go to a GP, or ready to see someone face to face. It’s a lot less invasive or intrusive or confronting to have an app tell you ‘hey, look, you’re really high risk. You really need to call Lifeline or you really need to…  Interviewer: Yeah, ‘we think you would really benefit from a little help and support’  Participant: Yeah yeah yeah. So that would hopefully add something where there was before nothing.” (*Interview 3*) |
| **Resistance to change**  “I think for some people it could be really helpful. Those who are motivated. Those who are proactive. I am thinking about certain personality types, there may be a reluctance to use it. I think it would really help some people. But I am assuming there would be a bunch, a handful who would want a professional to do those things for them.” (*Interview 1*) | **Training**  **“**Some sort of training that is accessible, probably free, that’s easy and not time consuming. So easy to get in, and…yeah.” (*Interview 2*) |
| **Not using the Platform as intended**  “The only thing I’m thinking of but I guess that’s always part of this, is people filling this in accurately and honestly and how easy it could be to get a concerning outcome. Like go see the GP and then going back and changing that to then not have that as a concern. Could that be an outcome. Could someone twist things to make it appear their okay to themselves? ” *(Interview 1)* | **Using the Platform as intended**  “I mean I guess the more that we offer it to people and more there may be an increase in people using the InnoWell platform, we might start to have more contacts and more people. I guess, clients contacting us who have been using it, and then who might be able to then talk to us in the lingo a little bit more. Because maybe, they’ve been tracking their mental health, and so like maybe the clients might become a little bit savvier overtime? (*Interview 3*) |
| ***Health professional (8 references)*** | ***Health professional (21 references)*** |
| **Digital Literacy**  “I think it’s just mainly clinicians need to be educated. So, as a rule, they have some sort of education and exposure to those things.” (*Interview 2)* | **Digital Literacy**  **“**Participant: **… j**ust reflecting on the technology changes that we’ve had in the last couple of months. I’d probably even be slightly into the early majority section.  Interviewer: A bit ahead of the game?  Participant: Yeah. In terms of, it’s been fairly easy for me to integrate the new technologies. Um…  Interviewer: Yeah, I might just channel down that a little bit. Why do you think you’re a little bit ahead?  Participant: I don’t know (laughs). Maybe an age thing. Maybe also that most of the new technologies have been fairly intuitive. So yeah… I mean in terms of like the learning and adopting, I can really see …you know, you get trained but then, being able to incorporate it is more just a trial and error. Get in there and have a play type of thing. And I guess I’m comfortable doing that then figuring out ways of doing things. Umm… yeah and it’s mostly been really intuitive so… I think that’s why. Also, because I’ve only been doing this role for a couple of months.” (*Interview 3*) |
| **Communication and team**  “It does rely a lot on communication in the room… there is no really way of doing that currently with technology in terms of giving yourself an amount of contacts that feels okay.” (*Interview 1)* | **Communication and team**  “It’s all shared. And I think that’s the culture. Because I think people might find stuff, and then we all tell each other about it and talk about it in a fairly open way. Rather than, this is my list and I’m not sharing it with anybody.” (*Interview 3)* |
| **Replacing clinical expertise**  I think with any technology or any change, that essentially is a positive thing, but there is always going to be something that is removed or changed by a result of that. So I am a but mixed on how that impacts on our role. Whether that will impact our need. But I guess it will enrich the counselling side of things perhaps. You know perhaps people will use our service more for counselling rather than information and resource. So yeah, it could work both ways. (*Interview 1)* | **Competency**  “Even if we’re on the phone, we’re looking, searching, taking down pieces of data from the call. Um, looking for referrals for people, and offering out resources and findings websites and self-help and this and that to be able to weave it into the conversation and pause as well.” (*Interview 3)* |
| **Scope of role**  “Interviewer: Do you try out new technology in your work?  Participant: In my work? No.  Interviewer: And why not?  Participant: I just think it’s not really the scope of my role. In terms of the time I’ve been at [the service] we did jump to a new technology, so yes, that’s happened once, but that’s been given to us by no choice. We’re moving to this new system.” (*Interview 1)* | **Readiness for change**  “I think they [technology solutions] can offer so much, they can make things so accessible, they can make it cheaper, they can kind of supplement a counselling practice, in so far as helping them support people ahead of the contact with the therapist. Like, use this app to track how you’re going all the time, or use it for your daily meditation, or whatever you do. It’s generally a really good thing for the industry, even though it might be a little disruptive.” (*Interview 3)* |
| ***Service (20 references)*** | ***Service (28 references)*** |
| **Funding**  “So the reason why we didn’t do many things – one of them was financial, and the other one is that it needs to get approved. So it’s like when I was talking about it. Even if people are keen, it takes time.” (*Interview 2)* | **Funding**  Interviewer: Is there anything else that really supports that [change towards adopting technology]?  Participant: (pauses) I guess the obvious one is that we’re quite well funded.  Interviewer: Yeah, right.  Participant: Yeah, I can see how if things were tighter in that area, then we might have to do things more quickly, or there might be pressure to cut corners or to spend less time with the experts who’ve been hired to create technological innovations for us. You know, there’s been plenty of time to kind of go back and forth with them. (*Interview 3)* |
| **Implementation**  “… and implementing it, from my opinion we are pretty over extended as it is. Perhaps not having enough people and knowledge to do it. Maybe not the technical things to implement the change I am imagining.” (*Interview 1)* | **Implementation, planning and training**  “…like if there’s a possibly for mental health care professionals to have an account as well, maybe to be kept up-to-date with resources or activities, yeah, I think that would be a good idea. That could be used for supervision.” (*Interview 2)* |
| **Resourcing**  “Interviewer: Are there particular barriers that you noticed, that get in the way … that make it hard for your clients to use technology in their care?  Participant: Yeah, I would say the structure of getting things approved. Then training. Particularly when it comes back to money and time. If we have time for team meetings, and money to cover it, you have so many things that you need to be trained in, that technology … if it’s not crucial, it will probably fall behind.” (*Interview 2)* | **Addressing service provision gaps**  “I think it would fill that gap quite well. Especially those who live regionally and waitlists, being able to get some clarity about where I am at can take time. Even just getting through to us can take time. So, if somebody can essentially get some sort of information about where they are at and what they are experiencing is of concern or in need of immediate, I think it would fill that gap nicely.” (*Interview 1)* |
| **Limits on clinical tool**  “I guess with the resources we recommend aren’t current, they’re not technology based, they’re more books, and they’re things you have to purchase and read. So you know going to see a therapist, physically, and having sessions, so all of that I would say, I mean I don’t know too much about what’s out there, but in that I’d say we are falling behind. We don’t share too much digital apps or what I imagine is out there. (*Interview 1)* | **Independent consultation and evaluation**  “Maybe something that can be assessed and experienced by non, people who are implementing it. So really using the technology. Perhaps people who aren’t in [the service] to actually assess what is the experience of using this and maybe gathering information from those who do use it to see, actually is it helpful and what is their experience.” (*Interview 1)* |
| **Existing IT and digital infrastructure**  “It’s just because… things haven’t been updated. [the] program that we use that’s pretty, um, old. It’s not that advanced and doesn’t have features that we probably need. It still has many things that still are not up to date. And even when that is brought up, due to the constraints, even when there’s desire to do it, it might not be approved. (*Interview 2)* |  |
| **Provision of training, support and professional development**  “I think both practitioners, especially our service needs to be updated on what’s available and use the things that are available, see if they’re good or not good. Kind of really assess them. So I think if we were more informed, and had more options of choices, then that may increase the clients willingness to know what’s available and engage.” (*Interview 1)* |  |
